# Capybara: Efficient estimation of generalized linear models with high-dimensional fixed effects

**DOI:** 10.1371/journal.pone.0331178

**Published:** 2025-09-16

**Authors:** Mauricio Vargas Sepulveda

**Affiliations:** Department of Economics, University of Surrey, Guildford, United Kingdom; Southwest Petroleum University, CHINA

## Abstract

This paper introduces capybara, an R package implementing computationally efficient algorithms for estimating generalized linear models (GLMs) with high-dimensional fixed effects. Building on Stammann (2018), we combine the Frisch-Waugh-Lovell (FWL) theorem with alternating projections to achieve memory-efficient estimation. Our benchmarks demonstrate that capybara reduces computation time by 95-99% compared to traditional dummy variable approaches while maintaining numerical accuracy to 5 decimal places. For a complex gravity model with 28,000 observations and 3,200 fixed effects, capybara completes estimation in just 6 seconds using 33 MB of memory, compared to 11 minutes and 12 GB with base R. The package is particularly valuable for trade economics, labor economics, and other applications requiring multiple high-dimensional fixed effects to control for unobserved heterogeneity, making previously infeasible models computationally tractable on standard hardware.

## Introduction

Fixed effects models are essential tools for controlling unobserved heterogeneity in panel data analysis. In trade economics, structural gravity models routinely require thousands of exporter-time, importer-time, and bilateral fixed effects [1]. Similarly, labor economics applications often involve worker, firm, and time fixed effects that quickly become computationally prohibitive with traditional estimation methods.

This article presents capybara, an R package that extends the alternating projections approach of [2], also describe in [3], to provide memory-efficient estimation of GLMs with k-way fixed effects. Our contribution is threefold: (1) we provide a user-friendly implementation that significantly reduces memory usage by leveraging an efficient use of the C++ language with the tested and efficient linear algebra routines from the Armadillo library [4,5]; (2) we demonstrate significant reductions in memory footprint and computation time compared to standard Iteratively Weighted Least Squares (IWLS) in R, Python, and Stata; and (3) we maintain numerical precision suitable for academic research and policy analysis.

The standard IWLS approach can fall short for structural gravity estimation. For context, some Poisson-Pseudo Maximum Likelihood (PPML) structural gravity model with three way exporter-time, importer-time, and exporter-importer fixed effects require around 12 GB of memory to obtain the estimated model coefficients, as we will detail in the benchmarks. The computational challenge is not merely one of patience, allowing a laptop to run overnight does not solve the fundamental constraint that memory represents a hard boundary. When estimation procedures require inverting matrices or storing intermediate results, memory requirements grow substantially, which can cause models to exhaust available RAM and render estimation unfeasible. It could be the case with importer-exporter-sector data such as agriculture, mining, energy, manufacturing, and services flows. Recent developments have addressed this challenge for linear models [2,3,6,7], and this work builds on these advances to provide memory-efficient routines for Linear Models (LMs) and Generalized Linear Models (GLMs) with high-dimensional fixed effects.

The remainder of this paper is organized as follows: describing the algorithmic approach to fitting GLMs with k-way fixed effects, explaining the software usage with the structural gravity model of trade, presenting comprehensive benchmarks, and providing a conclusion about the current implementation and future work derived from its limitations.

## Generalized linear models with K-way fixed effects

Consider a GLM with k-way fixed effects:


$$\eta =Z\gamma =D\alpha +X\beta =\sum_{k=1}^{K}D_{k}\alpha_{k}+X\beta$$

where *D*_*k*_ are dummy matrices for fixed effects categories, *X* contains variables of interest, and the expected outcome is $E(y)=\mu =h^{-1}(\eta )$ for link function h(·).

The computational challenge arises from the high-dimensional Hessian matrix. With thousands of fixed effects, direct computation of (*Z^T^WZ*)^−1^ can be unfeasible due to memory constraints.

Following [2], we adapt the FWL theorem to separate structural parameters from fixed effects in the Newton-Raphson update:


$$\gamma^{r}-\gamma^{r-1}=(Z^{T}W^{r-1}Z{)}^{-1}Z^{T}W^{r-1}\nu^{r-1}$$

This can be rewritten as a weighted regression:


$${\tilde{\nu }}^{r-1}={\tilde{D}}^{r-1}(\alpha^{r}-\alpha^{r-1})+{\tilde{X}}^{r-1}(\beta^{r}-\beta^{r-1})$$

where ${\tilde{W}}^{r}={\left(W^{r}\right)}^{1/2}$ and tildes denote weighted variables.

The key insight is that instead of computing the large projection matrix $M_{\tilde{D}}=I\;-\;\tilde{D}{\left({\tilde{D}}^{T}\tilde{D}\right)}^{-1}{\tilde{D}}^{T}$, we approximate it using alternating projections over individual fixed effects categories.

For each category *k*, the projection simplifies to:


$$(M_{{\tilde{D}}_{k}}v{)}_{i}=v_{i}-{\tilde{w}}_{i}\frac{\sum_{j\in g_{kj}}{\tilde{w}}_{j}v_{j}}{\sum_{j\in g_{kj}}w_{j}}\;\forall i\in g_{kj}$$

where *g*_*kj*_ denotes observations sharing level *j* in category *k*.

Adapting from the Newton-Raphson algorithm, we can iteratively update the parameters *β* and *η* until convergence for r=1,…,R iterations as in the following simplified algorithm:

**Algorithm 1. Alternating projections for GLM with high-dimensional fixed effects.**

1: Initialize $\beta^{0}$, $\eta^{0}$

2: Initialize *W*^(0)^ and $\nu^{\left(0\right)}$ based on initial estimates (model family specific)

3: **repeat**

4:   Compute weights *W*^(*r*−1)^ and working response $\nu^{\left(r-1\right)}$

5:   Center variables using alternating projections

6:   **for** each fixed effect category *k* **do**

7:    **for** each observation *i* **do**

8:     ${\tilde{X}}_{i}\leftarrow X_{i}-\frac{\sum_{j\in \text{same group as }i}w_{j}X_{j}}{\sum_{j\in \text{same group as }i}w_{j}}$

9:     ${\tilde{\nu }}_{i}\leftarrow \nu_{i}-\frac{\sum_{j\in \text{same group as }i}w_{j}\nu_{j}}{\sum_{j\in \text{same group as }i}w_{j}}$

10:    **end for**

11:   **end for**

12:   Repeat centering until convergence

13:   Solve for beta using transformed variables using Cholesky decomposition

14:   $\beta^{r}\leftarrow \beta^{\left(r-1\right)}+{\left({\tilde{X}}^{T}\tilde{X}\right)}^{-1}{\tilde{X}}^{T}\tilde{\nu }$

15:   Update linear predictor $\eta^{r}$

16: **until** convergence

17: Return $\hat{\beta }\leftarrow \beta^{r}$

For each group within each fixed effect category, we subtract the weighted group mean from each observation. By cycling through all fixed effect categories multiple times, we achieve the same effect as including thousands of dummy variables, but with minimal memory requirements. From the different alternatives to speed up the demeaning convergence, we used the Symmetric Kaczmarz method with a Conjugate Gradient acceleration [3,8].

The $\hat{\alpha }$ parameters for the fixed effects are recovered in a posterior step, using the estimated $\hat{\beta }$. This approach is a divide and conquer strategy that allows us to estimate models with thousands of fixed effects without running into memory issues and providing significant speedups compared to traditional methods at the same time.

## Software usage

Consider the following functional form for a PPML gravity model [1,9]:


$$X_{ijt}=\exp\left[\beta_{1}\log({\text{DIST}}_{ij})+\beta_{2}{\text{CNTG}}_{ij}+\beta_{3}{\text{LANG}}_{ij}+\beta_{4}{\text{CLNY}}_{ij}+\pi^{\text{OR}}+\pi^{\text{DE}}\right],$$

where:

- *X*_*ijt*_ = exports from country *i* to country *j* at year *t*
- ${\text{DIST}}_{ij}$ = distance between countries
- ${\text{CNTG}}_{ij}$ = common border dummy
- ${\text{LANG}}_{ij}$ = common language dummy
- ${\text{CLNY}}_{ij}$ = common colonial history dummy
- $\pi^{\text{OR}},\pi^{\text{DE}}$ = exporter-year and importer-year fixed effects.

Capybara computes the estimated slopes for this model as follows:



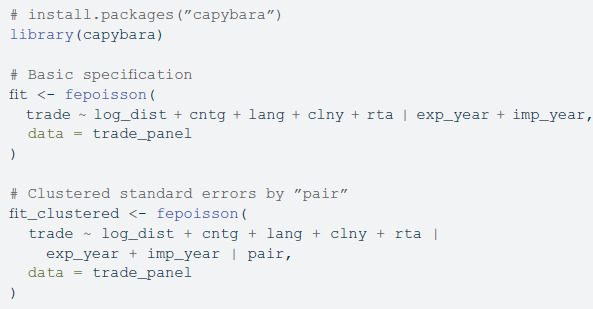



Table 1 presents estimation results for the gravity model:

**Table 1:** Estimation results for the PPML gravity model. Source: own creation.

Variable	Estimate	Regular SE	Clustered SE
log(dist)	–0.82	0.0004	0.0258
cntg	0.42	0.0004	0.0673
lang	0.25	0.0008	0.0623
clny	–0.21	0.0010	0.0914
rta	0.19	0.0010	0.0554

The results align with the intuition behind the gravity model [10]: trade decreases with distance and increases with common borders, common language, and trade agreements.

Furthermore, the summary() method provides a comprehensive overview of the model fit, including the number of observations, fixed effects, and convergence status. Table 2 and its footnote present the estimation results as returned by the summary() method:

**Table 2:** Summary results for the PPML gravity model. Significance codes: (***) 99.9%; (**) 99%; (*) 95%; (.) 90%. Pseudo *R*^2^: 0.587. Number of observations: 28,152. Source: own creation.

Variable	Estimate	Std. Error	*z* value	Pr(>|z|)
log(dist)	–0.8216	0.0004	–2194.0448	0.0000^***^
cntg	0.4155	0.0009	476.0613	0.0000^***^
lang	0.2499	0.0008	296.8884	0.0000^***^
clny	–0.2054	0.0010	–206.3476	0.0000^***^
rta	0.1907	0.0010	191.0964	0.0000^***^

In order to provide the pseudo *R*^2^ and the number of observations, capybara uses the methods described in [11], as the pseudo-*R*^2^ is defined as the squared Kendall’s *τ* between the observed and predicted values [9].

The fixed effects can be recovered using the fixed_effects() function (future versions will provide the fixed effects with the regression functions):



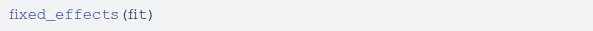



This returns a list of fixed effects for each category, which can be summarized as in Table 3:

**Table 3:** Partial view of the returned fixed effects. Source: own creation.

Country-Year	Importer FE	Exporter FE
ARG1986	9.57	10.03
ARG1990	9.59	10.90
ARG1994	11.30	11.08
ARG1998	11.67	11.55
ARG2002	10.40	11.49

Around seventy-percent of capybara’s code has been tested against base R IWLS, as it is relevant to determine the correctness of the results besides the performance gains [12].

### Benchmark

We obtained the estimated model coefficients for the following a three-way fixed effects PPML gravity model with roughly 28,000 observations and 3,200 fixed effects:


$$X_{ijt}=\exp[\beta_{1}{\text{RTA}}_{ij}^{t-12}+\beta_{2}{\text{RTA}}_{ij}^{t-8}+\beta_{3}{\text{RTA}}_{ij}^{t-4}+\beta_{4}{\text{RTA}}_{ijt}\;\;+\pi^{\text{OR}}+\pi^{\text{DE}}+\pi^{\text{DO}}+\pi^{\text{IN86}}+\pi^{\text{IN90}}+\pi^{\text{IN94}}\;\;+\pi^{\text{IN98}}+\pi^{\text{IN02}}],$$ (Globalization)

where:

- *X*_*ijt*_: exports from country *i* to country *j* at year *t*
- ${\text{RTA}}_{ijt}$: Regional Trade Agreement between countries *i* and *j* at time *t*
- ${\text{RTA}}_{ij}^{t+k}$: RTA between countries *i* and *j* at time *t* + *k*
- $\pi^{\text{IN86}},\pi^{\text{IN90}},\pi^{\text{IN94}},\pi^{\text{IN98}},\pi^{\text{IN02}}$: dummy variables taking the value of one for international trade for each year *Y*, and zero otherwise.
- $\pi^{\text{OR}},\pi^{\text{DE}},\pi^{\text{DO}}$: exporter-year, importer-year, and exporter-importer fixed effects

We compared the following implementations: base R IWLS (glm() with a Quasi-Poisson link) [13], fixest concentrated likelihood [7], and alpaca/capybara alternating projections [2,3]. The benchmarks used the same dataset and functional form, and results are summarized in Table 4 and Table 5.

**Table 4:** Benchmark median time (seconds) for different packages on the Globalization model. Ratio is relative to the slowest package (Base R, 100%). Source: own creation.

Package	Time (s)	Ratio (%)
Alpaca	6.4	0.9
Fixest	0.2	0.03
Capybara	0.7	0.1
Base R	700	100.0

**Table 5:** Benchmark memory allocation (MB) for different packages on the Globalization model. Ratio is relative to the largest allocation (Base R, 100%). Source: own creation.

Package	Memory (MB)	Ratio (%)
Alpaca	572	4.7
Fixest	78	0.6
Capybara	24	0.2
Base R	12,260	100.0

Key findings from the benchmark:

- Capybara completes estimation in 1.6 seconds using only 42 MB of memory, compared to Base R’s 700 seconds and 12,261 MB.
- This represents a reduction of over 99% in both computation time and memory usage relative to the standard R approach.
- While fixest achieves the fastest runtime at 0.3 seconds, Capybara provides the smallest memory footprint (0.3% of Base R), making it especially suitable for memory-constrained environments.
- These results highlight Capybara’s ability to efficiently estimate models with thousands of fixed effects, maintaining minimal memory usage even for highly complex specifications.

The benchmark was conducted on a Lenovo ThinkPad X1 Carbon Gen 9 laptop equipped with an 11th Gen Intel Core i7-1185G7 processor (8 cores, 3.00GHz), 15.3 GiB of RAM and Manjaro Linux operating system.

### Conclusion

Capybara provides an efficient solution for estimating generalized linear models with high-dimensional fixed effects, a major computational challenge in applied econometrics. Using a memory-efficient algorithm based on the Frisch-Waugh-Lovell theorem and alternating projections, capybara achieves substantial improvements over conventional methods and similar solutions.

The benchmark show that capybara reduces memory usage, making estimation feasible on standard laptops, even for models with a large number of fixed effects. Although packages like fixest may be faster in some cases, capybara lower memory usage makes it well-suited for large-scale or memory-constrained applications. It maintains numerical stability and offers fully open source solution for the R ecosystem. Future improvements to Capybara would consist in matching fixest speed while maintaining a minimal memory footprint.

Capybara is available on CRAN and GitHub, with documentation and examples covering bias correction methods. Extensive testing ensures its reliability as an econometric tool. The benchmarking script and results are available on GitHub for direct download.
